# Drug interference with biochemical laboratory tests

**DOI:** 10.11613/BM.2023.020601

**Published:** 2023-04-15

**Authors:** Jasmina Katanić, Bojan Stanimirov, Vanesa Sekeruš, Maja Đanić, Nebojša Pavlović, Momir Mikov, Karmen Stankov

**Affiliations:** 1 Department of Biochemistry, Medical faculty, University of Novi Sad, Novi Sad, Serbia; 2 Department of Pharmacology, Medical faculty, University of Novi Sad, Novi Sad, Serbia; 3 Department of Pharmacy, Medical faculty, University of Novi Sad, Novi Sad, Serbia

**Keywords:** biochemical marker, clinical laboratory test, diagnostic error, drug-laboratory test interaction, pharmaceuticals

## Abstract

Clinical laboratory practice represents an essential part of clinical decision-making, as it influences 60-70% of medical decisions at all levels of health care. Results of biochemical laboratory tests (BLTs) have a key role in establishment of adequate diagnosis as well as in evaluation of treatment progress and outcome. The prevalence of drug-laboratory test interactions (DLTIs) is up to 43% of patients who had laboratory results influenced by drugs. Unrecognized DLTIs may lead to misinterpreted BLTs results, incorrect or delayed diagnosis, extra costs for unnecessary additional tests or inadequate therapy, as all may cause false clinical decisions. The significance of timely and adequate recognition of DLTIs is to prevent common clinical consequences such as incorrectly interpreted test results, delayed or non-treated condition due to erroneous diagnosis or unnecessary extra tests or therapy. Medical professionals should be educated that it is essential to obtain patient data about medications especially for the drugs used in the last 10 days before biological material collection. Our mini-review aims to provide a comprehensive overview of the current state in this important domain of medical biochemistry with detailed analysis of the effect of drugs on BLTs and to give detailed information to medical specialists.

## Introduction

Laboratory testing is an essential part of clinical decision-making, influencing 60-70% of medical decisions at all levels of healthcare (*1*, *2*). Results of biochemical laboratory tests (BLTs) have a key role in establishment of adequate diagnosis (*3*-*6*). Biochemical laboratory tests ordering belongs to a very important pre-preanalytical phase (*7*, *8*). Increasing frequency in BLTs ordering requires that all health professionals be aware of the recent definition of appropriateness: “prescription of the Right test, using the Right method, at the Right time, to the Right patient, with the Right costs and for producing the Right outcome”, and medical tests should be consistent with clinical guidelines (*9*). Despite significantly improved knowledge on biomarkers generated from BLTs, knowledge of medical students and professionals about importance of extra-analytical phase of laboratory testing is not sufficient and requires constant improvement and education (*10*, *11*).

Laboratory practice is a cyclical process, typically divided in the preanalytical, analytical and postanalytical phase, where the preanalytical phase is considered as the most vulnerable part of the total testing process (*12*). Prevention of preanalytical errors and subsequent prevention of inappropriate treatment of patients due to incorrect test results, requires the permanent awareness of the primary factors linked to patient variables, sample collection and processing (*13*). Amongst patient characteristics, the most important are age, gender, feeding state, physiological changes and drug intake. All these patient-linked factors can modulate the results of BLTs by analytical or physiological interference with analysis. In Europe and worldwide there is a high prevalence of the use of over-the-counter (OTC) drugs, herbal preparations and dietary supplements that can significantly affect laboratory test results. The important survey in 18 European countries revealed that amongst 3600 patients, 68% were regularly taking at least one OTC drug or dietary supplement. In addition, in this large group of patients (N = 2429) taking at least one OTC drug or dietary supplement, 49% did not share this information with their physician. More detrimental, even amongst those who considered important to inform their responsible physician about consumption of OTC drugs and dietary supplements, 30% did not believe that they needed to disclose this information to laboratory staff (*14*). Thus, the results of this important study emphasize the significance of increasing the awareness of drug-laboratory test interactions (DLTIs) amongst patients, especially by their responsible physicians who may increase the perception of the patients that reporting the use of OTC drugs and dietary supplements improves the process of proper patient preparation for laboratory testing.

Even in an ISO15189 accredited laboratory that is certified to deliver valid and reliable examination results for their intended clinical use, laboratory results do not always correspond to the patient clinical status. Extra-laboratory factors, including fundamental procedures such as test requesting, which occur in pre-preanalytical phase, are error-prone and they account for 50-75% of all laboratory errors (*7*, *15*). Thus, it is of vital importance to recognize that BLTs offer value only if they are analytically and clinically valid, clinically relevant and cost effective (*16*). These qualities of BLTs may be significantly diminished by preanalytical errors, such as unrecognized influence of drugs on clinical laboratory results. Therefore, preanalytical error may occur when the BLT is ordered inappropriately, without adequate knowledge, information or correct interpretation of DLTIs (*17*, *18*).

Our mini-review aims to provide a comprehensive overview of the current state in this important domain of medical biochemistry. The methodology that we used in the systematic literature search for Supplementary table data is the analysis of keywords: medicine, drug, laboratory, test, interaction, interference, cholesterol, lipoprotein, triacylglycerol, triglyceride, glucose, bilirubin, urate, uric acid, creatinine, aminotransferase, CRP. The search strategy was adapted as needed and a hand-search of articles from relevant reviews was conducted to identify studies for potential inclusion. The detailed analysis of 367 references is summarized in Supplementary table 1, in which the prescription drugs are listed according to Anatomical Therapeutic Chemical (ATC) Classification, with the most common biochemical laboratory parameters that are modified by each particular drug.

## Drug-laboratory test interactions

Results of numerous routinely performed and highly specialized BLTs in serum and other biological material can be influenced by one or more drugs that are prescribed to patients. Therefore, DLTIs represent an important source of diagnostic and/or therapeutic errors (*19*). This emphasizes the importance of knowledge and continual education regarding the possible DLTIs, for medical doctors, pharmacists and laboratory specialists (*20*).

Polypharmacy (polypragmasia) is defined as the concurrent use of five or more medications (*21*). The prevalence of polypharmacy in adults aged 65 years or more ranges from 26.3-39.9% across 17 European countries plus Israel, with the lowest prevalence of polypharmacy in Switzerland, Croatia and Slovenia (26.3%, 27.3% and 28.1% respectively), and highest prevalence in Portugal, Israel and Czech Republic (36.9%, 37.5% and 39.9% respectively) (*22*). Polypharmacy is associated with increased risk of occurrence of drug-related problems, including drug-laboratory test interactions and adverse health outcomes (*23*). In addition to polymedication and presence of comorbidities in elderly, age-related physiological changes of hepatic and renal function are implicated in altered drug pharmacokinetics. All these factors significantly contribute to complex and careful drug therapy in the elderly and need for improved knowledge about DLTIs (*23*). Medical professionals should be educated that it is essential to obtain patient data about medications as well as the timing of drug used by the patient within 10 days before collecting biological material for the correct conduction and interpretation of a laboratory test (*24*).

The importance of drug-drug interactions that can lead to serious unwanted effects or to a reduction in the therapeutic effects is well recognized in medical practice. Mechanisms of drug interactions with endogenous molecules in body fluids and tissues, with laboratory test components or with other drugs in case of polypharmacy are numerous, but they can all be classified into pharmacokinetic (PK) and pharmacodynamic (PD) mechanisms of interactions (*25*).

For PK interactions, the ADME (absorption, distribution, metabolism and excretion) principle describes the interactions at the level of proteins responsible for the disposition of drugs. After peroral intake, drug absorption in enterocytes occurs through passive or facilitated diffusion and it is controlled by the presence of drug metabolizing enzymes and drug transporters that can be either induced or inhibited by drugs (*25*). Numerous drugs (*e.g.* statins or macrolides) affect absorption of drugs by inducing or inhibiting drug transporters such as efflux ATP-binding cassette (ABC) and uptake solute carrier (SLC) families of transporters in the apical and basolateral membrane of enterocytes. In addition, duodenal enterocytes express microsomal enzymes that belong to cytochrome P450 (CYP) enzyme family (CYP3A and CYP2C subfamily), which can be induced or inhibited by drugs, thus modifying the bioavailability of drugs and their interactions (*25*).

Upon absorption into the systemic circulation, the distribution to tissues can occur by passive diffusion and membrane protein-mediated transport. Drugs interact with plasma proteins and binding of drugs to plasma proteins is one of many factors that determines drugs’ ADME (*25*). Binding of drugs occurs at the level of multiple blood constituents such as albumin, α1-acid glycoprotein, lipoproteins, red blood cells, leukocytes, platelets and α-, β- and γ-globulins. Binding between drugs and plasma proteins is usually reversible, due to weak hydrophobic and electrostatic interactions such as van der Waals and hydrogen bonding (*25*). Complexes between drugs and plasma proteins in the blood plasma serve as drug reservoirs for the free drug concentration, which affects drug distribution, elimination, the efficacy of the drug and/or its possible toxicity. Interactions between drugs and plasma proteins, especially the binding percentage of the drug with albumins may be affected by co-administration of other drugs or nutrients. One of the best-known examples of competitive displacement of bilirubin from albumin by sulphonamides and subsequent jaundice is described in neonates and with ibuprofen, diazepam, cyclosporine and salicylates use. Due to decreased affinity of albumin for bilirubin in neonatal period, the bilirubin may be displaced from its binding site in albumin by drugs, resulting in clinical jaundice (*26*).

Drug interactions with other endogenous and/or exogenous molecules due to enhanced metabolism by induction or allosteric activation of CYP enzymes may have clinical consequences. In addition, numerous drugs are CYP inhibitors (competitive, non-competitive, and mechanism-based) and these interactions have a particular importance that require specific clinical management strategy (*27*).

The biochemical interactions of drugs with body molecules are described as PD response. Interactions with other drugs may induce additive, synergistic or antagonistic PD response. Interactions with BLTs are classified as a pharmacological type of DLTIs (*28*, *29*).

Timely and adequate recognition of significant DLTIs is critical to prevent common clinical consequences such as incorrectly interpreted test results, delayed or non-treated condition due to erroneous diagnosis, unnecessary extra tests, or inadequate therapy. The distribution of interactions by clinical importance according to comprehensive recent review, positions antibacterial agents, specifically cephalosporins, as the most frequently reported drugs that affect the BLTs (glucose and creatinine in blood) (*29*). Amongst other FDA-approved drugs that interfere with laboratory results, the second most frequent DLTIs are those that appear in patients taking psychotropic drugs, such as antidepressants, antidyskinesia agents and antipsychotic drugs. In these patients, the most frequent DLTIs are false positivity for ketone bodies and false negativity for glucose in urine, as well as false positivity/elevation for phenylketonuria test and pregnancy test results in blood. Other clinically important DLTIs comprise those induced by contrast media (proteinemia, bilirubin, creatinine, iron, calcium, coagulation factors); by proton-pump inhibitors (higher serum concentrations of chromogranin A) and acetaminophen interference with continuous glucose monitor (CGM) sensing, which results in erroneously high readings (*29*). Several CGM devices are designed to eliminate interference from acetaminophen, but there is still important interference from ascorbic acid and antineoplastic drug, hydroxyurea (*30*).

An extensive on-line database of the effects of drugs, disease, preanalytical variables, and herbals on laboratory tests, created by principal editor DS Young, contains information on more than 135,000 effects on more than 5,000 tests, with > 50,000 DLTIs (*19*, *31*). Table 1 presents the selection of the most useful DLTIs databases with internet addresses. The prevalence of DLTIs is variable and depending on the hospital ward, literature reports high prevalence of up to 43% of patients who had laboratory results influenced by drugs (*32*, *33*).

**Table 1:** Selection of DLTIs databases with internet addresses

**Database**	**Web address**	**Accessed**
AACC Effects on Clinical Laboratory Tests (John Wiley and Sons, Inc., on behalf of the American Association for Clinical Chemistry)	https://clinfx.wiley.com/aaccweb/aacc/login	Feb 3^rd^ 2023
First DataBank MedKnowledge Database. Hearst Health Network	https://www.fdbhealth.com/solutions/medknowledge-drug-database	Feb 3^rd^ 2023
Dailymed database (The National Library of Medicine (NLM), a National Institutes of Health (NIH) institute)	https://dailymed.nlm.nih.gov/dailymed/	Feb 3^rd^ 2023
Exeter Clinical Laboratory. Blood Sciences department at the Royal Devon & Exeter NHS Foundation Trust, UK.	https://www.exeterlaboratory.com/blood-sciences/	Feb 3^rd^ 2023
Drug effects in clinical chemistry (the Swedish Society for Clinical Chemistry in collaboration with the National Corporation of Pharmacies)	https://www.tryding.se/	Feb 3^rd^ 2023
Multirec (Multirec Ltd, Turku, Finland)	https://www.multirec.fi/products/mr-dle/	Feb 3^rd^ 2023

Consequences of unrecognized DLTIs as preanalytical variables may significantly disturb the analytical process and postanalytical phase. Results of BLTs may be misinterpreted and lead to incorrect or delayed diagnosis, to extra costs for unnecessary additional tests or inadequate therapy, all of which have an important negative clinical impact (*34*). Linking the laboratory and pharmacology, BLTs and drugs prescriptions, presents an important approach to improve the utilization and quality of both laboratory testing and pharmacotherapy, as well as to provide opportunities for improved outcomes and learning (*35*, *36*).

The main concern of health providers regarding the patients’ safety is to reduce diagnostic errors (defined as incorrect, missed, or delayed diagnoses) that may be the consequence of miscommunication, misinterpretation and missing results (*37*). In clinical laboratories settings, the basis for accurate laboratory tests and improved quality of all phases of the testing process is the implementation of the laboratory information system (LIS).

One of the first LIS that includes patients’ medication data was described more than 25 years ago (*38*). Laboratory information system is a key component of a successfully implemented electronic health record (EHR), which combines the clinical documentation module that captures the patient’s clinical data such as diagnosis, procedure, complication and medication (*39*). In secondary and tertiary health care, the implementation of a LIS that is integrated with an EHR, significantly improved within-laboratory turnaround time, decreased test requests and preanalytical errors, while increasing efficiency and improving provider satisfaction. In addition to improved quality of patient care and reduced errors, LIS links communication between clinical and laboratory medical services. The laboratory information system is an integral part of laboratory data and process management and includes automatic monitoring and evaluation of potential effects of drugs on laboratory tests (*40*). In modern laboratories, the analytical phase represents the component of the testing process that is the least error-prone (only 15% of all mistakes), due to highly automated and standardized support provided by a LIS (*41*).

## Classification of DLTIs

Drug-laboratory test interactions fall into two broad categories: physiological (pharmacological, biological, *in vivo*) and analytical (methodological, *in vitro*) interference (*18*).

The first type of DLTIs is the most frequent category that refers to the influences of drugs and their metabolites on BLTs, which are independent of the BLT method used in laboratory. This physiological type of DLTIs can be identified when the change of laboratory parameter under the influence of drug is expected (the intended effects of drugs) (*19*). An illustrative example of this is the decrease of thyroid stimulating hormone upon thyroid hormone replacement therapy (*19*, *42*). On the other hand, the identification of DLTI is difficult in case of unwanted or toxic drug effects such as idiosyncratic drug reactions (IDR) (*43*). Such DLTIs may lead to wrong, missed or delayed diagnosis, which is the definition of diagnostic error (*44*).

Recently published data indicate that additional unnecessary diagnostic procedures are carried out due to DLTI, which is well known in medical practice (*19*). In patients with neuroendocrine tumors (NETs), standard of care requires testing of the most important biochemical tumour markers: chromogranin A (CgA, diagnostic sensitivity and specificity within the range of 60-90%) and neuron-specific enolase (NSE) (*45*). In addition to the higher circulating CgA concentrations that have been demonstrated in serum or plasma of patients with different NETs, the stimulated CgA release is also possible from secretory granules of gastric enterochromaffin-like cells (ECLs) in non-NET patients who used proton pump inhibitors (PPIs). Even the short-term application of PPIs for 7 days stimulates hyperplasia of gastric ECLs, in which PPIs-induced gastrin elevation enhances the transcription of gene coding for CgA protein. Thus, in clinical practice, the use of PPIs is the most common cause of false (non-NET) CgA increase, and PPIs need to be discontinued for at least 14 days before a CgA test (*46*). Clinical and laboratory specialists need better awareness of this physiological type of DLTI. The results of an important retrospective study revealed the additional costs and discomfort for patients due to unnecessary diagnostic work-up, which was performed as a consequence of CgA and PPI interaction. Repeated CgA measurement (until CgA concentrations were normalized upon PPIs discontinuation) and somatostatin receptor PET imaging could have been avoided. Additional importance of this DLTI is underlined by high prevalence of NET (57%) in patients with both elevated CgA and prescribed PPIs (*34*).

Analytical (or *in vitro*) interactions between drugs and results of BLTs increase the chance of errors in the laboratory analytical process and important clinical consequences. Thus, adequate knowledge of such DLTIs may prevent errors in test interpretation, while the avoidance can be achieved by selection of an appropriate laboratory test method that is not influenced by drugs. Important examples comprise analytical DLTIs with commonly used drugs and BLTs that are used to guide the clinical decisions. There is an increase of up to additional 200 μmol/L of creatinine due to positive interference of cephalosporins (excluding cefotaxime and ceftazidime) with the Jaffe analytical method for creatinine (cefoxitin at concentrations ≥ 100 µg/mL for up to 2 hours post-infusion) (*47*). Falsely higher blood glucose values are determined by capillary blood glucose meters, in patients receiving intravenous vitamin C therapy (*48*). High-dose intravenous vitamin C (6 g/day for ≥ 5 days) is associated with lower mortality in patients with severe sepsis and septic shock (*48*). The cornerstones of therapy for patients in septic shock are adequate hemodynamic resuscitation, vasopressor therapy, and ventilation support, and in these patients high-dose vitamin C therapy decreases the fluid and vasopressors requirements (*49*). However, the well-known interference between vitamin C and glycaemia measurement method may result in erroneously recognized pseudohyperglycaemia and improperly indicated insulin therapy, leading to dangerous, possibly even fatal consequences. Since the hexokinase spectrophotometric method does not interfere with vitamin C, its use is recommended for point-of-care glucose monitoring in patients receiving intravenous high-dose ascorbic acid therapy (*49*). Considering the ascorbate PK, glycaemia measurements should be performed for at least 8-10h after intravenous vitamin C therapy (*50*).

Numerous important examples of DLTIs are described in our literature survey presented in Supplementary table 1, in which we provided a comprehensive and relevant list of interactions between drugs and BLTs, which may improve the knowledge of healthcare providers, including physicians, pharmacists and laboratory specialists. In Table 2, we selected several examples of the most commonly prescribed drugs and DLTIs. Within the limitations of our comprehensive review, we directly consulted and analysed only the primary sources *i.e.*, the scientific articles published in PubMed, however for construction of Supplementary table 1 we did not consult several DLTIs databases that also provide the overview of interactions and the corresponding available literature.

**Table 2:** Selection of most commonly prescribed drugs and DLTIs

**Drug**	**Laboratory parameter**	**Change**	**Mechanism**
Ascorbic acid	Total cholesterol	Decrease	Negative interaction with Trinder’s reaction
	Triglycerides	Decrease	
	Uric acid	Decrease	
	Creatinine	Increase	Positive interaction with Jaffe reaction
	Total bilirubin	Decrease	/
Acetaminophen	Glucose	Increase	Falsely elevated continuous glucose monitor (CGM) sensing
Cefalotin	Creatinine	Increase	Positive interaction with Jaffe reaction
Cefazolin			
Cefpirome			
Ceftriaxone	Total bilirubin	Increase	Competitive binding to albumin
Ciprofloxacin	Glucose	Increase/decrease	Impaired glucose homeostasis
Levofloxacin			
Moxifloxacin			
Gatifloxacin			
Fluoxetine	Triglycerides	Increase	/
Isoflurane	Total bilirubin	Increase	/
Sevoflurane			
Ritonavir	Triglycerides	Increase	Increased production of very low-density lipoprotein
Lopinavir			
Atazanavir			
Darunavir			
Simvastatin	Glucose	Increase	Impaired insulin secretion, impaired glucose uptake by the cells
Atorvastatin			
Rosuvastatin			
Warfarin	Uric acid	Increase	Enhanced uric acid production

## Concluding remarks

In conclusion, we believe that synergy of advanced analytical methods and interdisciplinary researchers as well as improved digitalization and automation of laboratory medicine with implementation of artificial intelligence into analysis of complicated medical data, is the pathway for improved precision and continuous optimization of the laboratory processes. This is an opportunity to advance healthcare with very important improvements for patients, but also for health professionals, data scientists, engineers and analytical chemists, with the main aim to reduce the prevalence of incorrect diagnosis, inadequate treatment and unnecessary follow-up due to DLTIs.
